# The Influence of Music Tempo on Inhibitory Control: An ERP Study

**DOI:** 10.3389/fnbeh.2020.00048

**Published:** 2020-05-07

**Authors:** Rong Xiao, Cuihong Liu, Jiejia Chen, Jie Chen

**Affiliations:** ^1^School of Educational Science, Hunan Normal University, Changsha, China; ^2^Cognition and Human Behavior Key Laboratory of Hunan Province, Hunan Normal University, Changsha, China

**Keywords:** music tempo, inhibitory control, Go/No-go paradigm, N2, P3

## Abstract

The purpose of the present study is to investigate the influence of music tempo on inhibition control. An electroencephalogram (EEG) was recorded when participants performed a Go/No-go task while listening to slow (54 bpm), medium-paced (104 bpm), fast (154 bpm), or no music. The behavioral results showed that the accuracies for the No-go trials were lower in the fast than in the slow tempo music conditions, while the accuracies for the Go trials were also lower in the fast tempo than in no music conditions. The event-related potential (ERP) study results showed that larger N2 and P3 amplitudes were elicited by No-go than by Go conditions. Moreover, the difference N2 (N2d) amplitudes observed by No-go vs. Go condition were larger in fast music than in medium-paced, slow, and no music conditions, indicating more consumption of cognitive resources in the process of conflict monitoring under the fast music condition. However, no such differences were observed among medium-paced, slow, and no music conditions. In addition, the difference P3 (P3d) amplitudes, an index of response inhibition, were not significant among these four music conditions. The present study showed a detrimental influence of music tempo on inhibition control. More specifically, listening to fast music might impair an individual’s ability to monitor conflict when performing the inhibitory control task.

## Introduction

The popularity of music in the field of psychological research has been increasing. More and more researchers regard music as the product of a general-purpose cognitive architecture and then discuss it from different perspectives of musical elements (e.g., mode, rhythm, tempo, etc.; Sutton and Lowis, 2008; Levitin et al., 2018; Navarro et al., 2018). An investigation into these elements of music not only has strong operability and practical significance but also is the basis for our understanding of the effects of music on human cognition.

Music tempo, which is measured in terms of beats per minute (bpm), is a representative of the basic dimension of music (Karageorghis et al., 2011). Moreover, it has been found that the tempo of music can affect not only human’s cognition such as attention, time perception, decision-making (North et al., 1998; Amezcua et al., 2005; Day et al., 2009), but also human’s consumption, diet, or driving behaviors. For example, it was found that participants made faster stimulus evaluation and response in fast than in slow tempo music conditions during a visual selective attention task (Amezcua et al., 2005). The decision accuracy was also higher in fast than in slow tempo music conditions during a multi-attribute decision-making task (Day et al., 2009). Moreover, previous studies have shown that the in-store traffic in a supermarket could be speeded up and the daily gross sales volume increased when the background music played in fast tempo relative to that played in slow tempo (Milliman, 1982). Furthermore, the background music of fast tempo could shorten restaurant patrons’ dining time (Milliman, 1986), with drinking speed increased (McElrea and Standing, 1992). Brodsky (2002) investigation into the impact of music tempo on simulated driving performance and vehicle control showed that the fast-paced music would increase the simulated driving speed and perceived speed estimate. Moreover, vehicular collision, lane crossings, and disregarded red traffic lights were more frequent during simulated driving in fast-paced than in low-paced background music conditions. Brodsky (2002) suggested that fast music could consume a driver’s attentional resources and impaired their motor control.

Actually, most of our daily activities, such as consumption, shopping, diet, or driving behaviors as mentioned above, are associated with human’s executive functions (also called cognitive control; Burkhard et al., 2018). Moreover, previous studies have demonstrated a close relationship between executive functions and musical training (Zuk et al., 2014). However, much less is known about the influence of music tempo on executive functions. Given the considerations mentioned above, the present study aimed to investigate the influences of music speed on executive functions. More specifically, we adopted event-related potentials (ERPs) and Go/No-go paradigm to investigate the temporal features underlying the influences of music speed on inhibition control. As an important subcomponent of executive functions, inhibition control is the ability to suppress inappropriate thoughts and responses (Diamond, 2013). Inhibitory control is frequently measured by using the Go/No-go paradigm, in which subjects were asked to respond to the “Go” stimulus and withhold their responses to the “No-go” stimulus (Falkenstein et al., 1999; Luijten et al., 2011).

Thus, in the present study, an electroencephalogram (EEG) was recorded when the participants performed the Go/No-go task while listening to slow (54 bpm), medium-paced (104 bpm), fast (154 bpm), or no music. Moreover, we put our focus on two ERP components, N2 and P3, both of which have been widely observed in the Go/No-go task. Specifically, the N2 amplitudes were larger for No-go trials relative to Go trials, reflecting the process of conflict monitoring (Nieuwenhuis et al., 2003). Moreover, the P3 amplitudes were also larger for No-go trials relative to Go trials, indexing the process of response inhibition (Falkenstein et al., 1999). In order to highlight the No-go N2 and the No-go P3 effects, the difference N2 (N2d) and P3 (P3d) waveforms were observed by subtracting the Go from the No-go conditions (Falkenstein et al., 1999; Gajewski and Falkenstein, 2013; Burkhard et al., 2018). Thus, we aimed to explore whether or not music tempo could affect the inhibitory control as evidenced by behavioral and neural indices. If music tempo influenced the inhibitory control, then different Go and No-go accuracies as well as the N2d and P3d amplitudes would be expected among music of different tempos. Otherwise, no behavioral and neural differences would be observed.

## Materials and Methods

### Participants

To establish the sample size, *a priori* statistical power analysis for a repeated-measures design was conducted using G*Power 3.1.9.2 (Faul et al., 2007). According to the software, a total sample size of *n* = 19 would be required to obtain a medium effect size of Cohen’s *f* = 0.25 (*a* = 0.05, power = 0.8; Cohen, 1988). To ensure a sufficient number of participants, a sample size of 26 participants (10 females, mean age = 19.5 years, SD = 1.4) were selected in the present study. All subjects were right-handed, with normal or corrected-to-normal visual acuity and no history of neurological diseases or color blindness. Ethical approval for the study was obtained from the Research Ethics Committee of the Hunan Normal University. The participants also signed an informed consent form before the experiment and were given appropriate rewards upon completion of the experiment.

### Materials

The first movement of Beethoven’s “Moonlight Sonata” was selected at the original 54 bmp for slow tempo musical excerpt. Similar to the previous studies (Brodsky, 2002; Bishop et al., 2014), this original musical excerpt was recomposed to 104 bmp for the medium and 154 bmp for the fast musical excerpts using the Adobe Audition CS6 (Adobe Systems Inc., San Jose, CA, USA) software. All the participants in this experiment are not familiar with these three musical excerpts. Dynamic earphones (Air Pods 2) with noise cancellation function were used for the participants to listen to the music. In addition to these three music conditions, there is also a no music condition, in which the participants performed the Go/No-go task with no audio input. The music loudness value is set to 70 dB SPL, which could be adjusted by the subjects at will to ensure maximum comfort.

### Procedure

**Figure 1:**
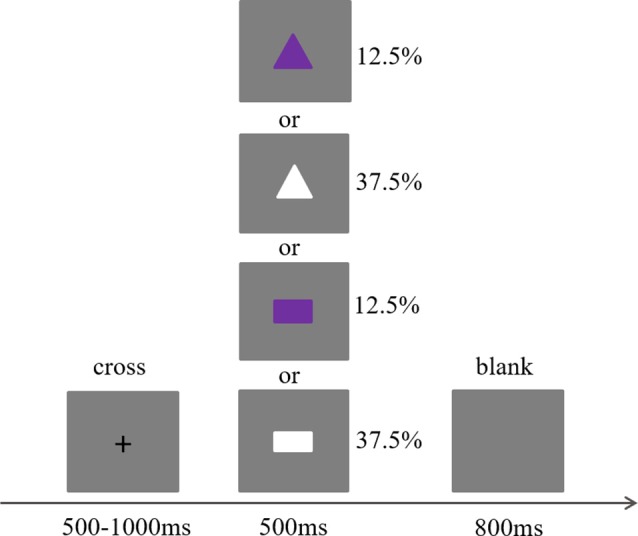
Task parameters for the Go/No-go paradigm. The task was presented using E-Prime v. 2.0 (Psychology Software Tools Inc., Pittsburgh, PA, USA) software running on an IBM-compatible computer. The presentation of trials was randomly switched, and each subject was required to press the buttons when presented go (white rectangle or white triangle) trials and give no response to No-go (purple rectangle or purple triangle) trials.

This study adopted the Go/No-go paradigm, which is a classical paradigm to investigate inhibition control (Diamond, 2013). The stimuli in this task were two kinds of shapes with different colors: a white rectangle, a purple rectangle, a white triangle, and a purple triangle. All the white stimuli were Go trials (75%) and all the purple stimuli were No-go trials (25%), with each type of stimulus presented randomly. Each trial was initiated by a small black cross presented for a duration ranging from 500 to 1,000 ms. Afterwards, one of the four types of stimuli was presented for 500 ms, which then was followed by a gray screen presented for 800 ms (see Figure 1). The participants were required to press a key on Go trials and not to press a key on No-go trials while listening to slow tempo, medium tempo, fast tempo, and no music. Thus, the present study included four blocks (the slow tempo, medium tempo, fast tempo, and no music blocks). Each block contained 240 trials (180 Go and 60 No-go trials), and the order of these four blocks was balanced across the participants. At the end of each block, a self-reported rate of this music was required on a scale of 1–9 in terms of induced pleasure (unpleasant to pleasant), arousal (calm to intense), and preference (dislike to like). After the rating, there was also a break of at least 5 min.

### Data Recording and Processing

An EEG was recorded from 64 scalp sites using tin electrodes mounted in an elastic cap (Neuro Scan Inc.,) with an online reference to the CPz. During the offline analysis, the EEG was re-referenced to the average of the right and the left mastoids. All interelectrode impedances were maintained under 5 KΩ. The EEG signals were amplified with a 0.1–30-Hz bandpass filter and were continuously sampled at 500 Hz/channel. The EEG was averaged in 800 ms epochs (200-ms baseline) that were time-locked to the presentation of the stimulus mark. According to previous ERP literatures regarding the Go/No-go task (Huster et al., 2010) and through the inspection of the topographic maps and grand-averaged ERP waveforms, we analyzed two specific components, N2 (260–320 ms) and P3 (400–500 ms) with the following regions: frontal (F3, F1, Fz, F2, and F4), fronto-central (FC3, FC1, FCz, FC2, and FC4), central (C3, C1, Cz, C2, and C4), centro-parietal (CP3, CP1, CPz, CP2, and CP4), and parietal (P3, P1, Pz, P2, and P4) regions. A three-way repeated analysis of variance (ANOVA) was conducted on the mean amplitudes of N2 and P3, with music tempo (four levels: 54 bpm, 104 bpm, 154 bpm, and no music), stimulus type (Go and No-go trials), and brain regions (five levels: frontal, fronto-central, central, centro-parietal, and parietal) as within-subject factors. The difference N2 and P3 waveforms were observed by subtracting the Go from the No-go conditions. In addition, one-way ANOVA was conducted on the behavioral accuracy and reaction times (RTs) with music tempo as within-subject factor. The degrees of freedom of the F-ratio were corrected by Greenhouse–Geisser. False discovery rate correction was applied for *post hoc* multiple comparisons.

## Results

### Behavioral Results

The ANOVA for No-go accuracy showed a significant main effect of music tempo (*F*_(3,75)_ = 4.48, *p* = 0.017, $\eta_{\text{p}}^{2}$ = 0.15). The *post hoc* multiple comparisons revealed that the accuracies were lower in fast than in slow music conditions. The ANOVA for Go accuracy (*F*_(3,75)_ = 8.93, *p* = 0.001, $\eta_{\text{p}}^{2}$ = 0.26) and RT (*F*_(3,75)_ = 42.43, *p* < 0.001, $\eta_{\text{p}}^{2}$ = 0.71) also showed a significant main effect of music tempo. The accuracy in the no music condition was higher than those in the fast-paced (*p* = 0.006), the medium-paced (*p* < 0.001), and the slow-paced (*p* = 0.006) music conditions. RTs in the fast-paced music condition were shorter than those in the medium-paced and in the slow-paced music conditions (*p*s < 0.004), in which RTs were also shorter than those in the no music condition (*p*s < 0.001; Table 1).

**Table 1:** Results of the one-way repeated-measures analysis of variance (ANOVA) for the accuracy of Go and No-go trials and the reaction time (RT) of Go trials.

Conditions	Fast (154 bpm)	Medium-paced (104 bpm)	Slow (54 bpm)	No music	*F*
	Mean (SD)	Mean (SD)	Mean (SD)	Mean (SD)	
Accuracy of No-go trials (%)	93.78 (0.06)	95.32 (0.03)	96.35 (0.03)	96.28 (0.04)	4.48*
Accuracy of Go trials (%)	94.64 (0.08)	95.86 (0.04)	95.23 (0.07)	99.25 (0.02)	8.93**
RT to Go trials in ms	333.10 (24.67)	340.76 (23.24)	346.91 (25.85)	376.10 (38.19)	42.43***

Notes: *p < 0.05; **p < 0.01; ***p < 0.001.

In addition, the ratings on music-induced pleasure, arousal, and preference showed no significant main effects on arousal (*F*_(2,50)_ = 1.78, *p* = 0.18, $\eta_{\text{p}}^{2}$ = 0.07) and preference (*F*_(2,50)_ = 2.34, *p* = 0.11, $\eta_{\text{p}}^{2}$ = 0.086). A significant main effect on pleasure was observed (*F*_(2,50)_ = 5.93, *p* = 0.005, $\eta_{\text{p}}^{2}$ = 0.19), with higher scores for medium than for slow tempo musical excerpts (*p* = 0.006). However, no significant differences were observed between medium and fast (*p* = 0.19) or slow and fast (*p* = 0.08) tempo musical excerpts.

### ERP Results

**Figure 2:**
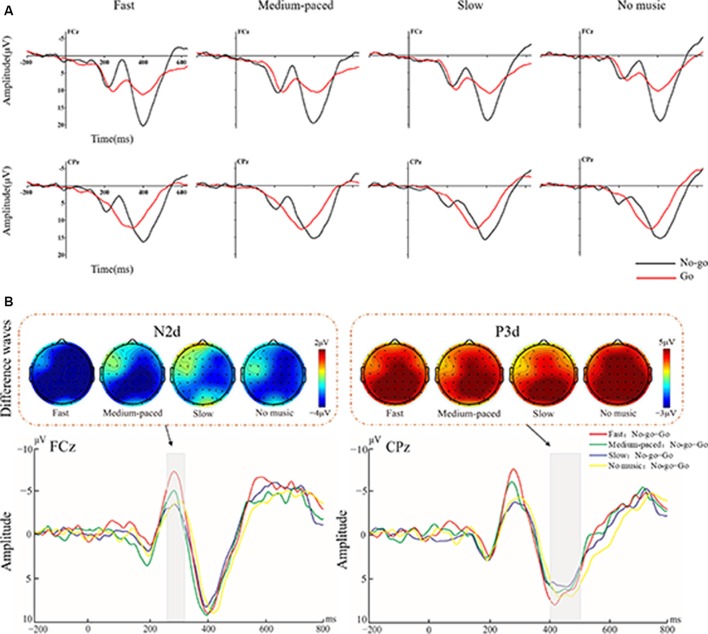
**(A)** Averaged event-related potentials (ERPs) at electrode FCz and CPz for Go (red lines) and No-go (black lines) trials separated by conditions of music tempo (fast, medium-paced, slow, and no music). **(B)** The different waves (No-go minus Go) at FCz and CPz for the different tempos are shown in the lower panel. The amplitudes of the N2d and P3d components for tempos are indicated by different line colors: fast, red lines; medium-paced, green lines; slow, blue lines; and no music, yellow lines. The topographical maps of the N2d (left panel) and P3d (right panel) components for different tempos are shown in the upper panel.

The ANOVA for N2 amplitudes showed a significant main effect on stimulus type (*F*_(1,25)_ = 50.35, *p* < 0.001, $\eta_{\text{p}}^{2}$ = 0.67), and the No-go condition elicited more negative N2 than the Go condition (see Figure 2A). Moreover, the interaction between stimulus type and music tempo was significant (*F*_(3,75)_ = 4.95, *p* = 0.005, $\eta_{\text{p}}^{2}$ = 0.17). The difference N2 amplitudes, obtained by subtracting the Go from the No-go conditions, were larger in the fast-paced music condition (−5.19 μV) than those in the medium-paced (−3.48 μV, *p* = 0.05), slow-paced (−2.51 μV, *p* < 0.001), and no music (−3.16 μV, *p* = 0.045) conditions. However, no significant differences were observed among the medium-paced, slow-paced, and no music conditions (*p*s > 0.26). The interaction between stimulus type and brain region was also significant (*F*_(4,100)_ = 4.88, *p* = 0.02, $\eta_{\text{p}}^{2}$ = 0.16). The N2d amplitudes were largest at the centro-parietal region. In addition, there was no significant interaction effect among music tempo, stimulus type, and regions (*F*_(12,300)_ = 1.88, *p* = 0.13, $\eta_{\text{p}}^{2}$ = 0.07).

The ANOVA for P3 amplitudes showed a significant main effect on stimulus type (*F*_(1,25)_ = 54.14, *p* < 0.001, $\eta_{\text{p}}^{2}$ = 0.68), and the No-go condition elicited larger P3 amplitudes than the Go condition (see Figure 2B). The interaction between stimulus type and brain region was significant (*F*_(4,100)_ = 7.13, *p* = 0.008, $\eta_{\text{p}}^{2}$ = 0.22). The P3d amplitudes were largest at the parietal region. However, no significant interaction effects were observed between stimulus type and music tempo (*F*_(3,75)_ = 1.005, *p* = 0.39, $\eta_{\text{p}}^{2}$ = 0.04) and among stimulus type, music tempo, and brain region (*F*_(12,300)_ = 1.53, *p* = 0.2, $\eta_{\text{p}}^{2}$ = 0.06).

## Discussion

The current study examined the influences of different tempos of music on inhibitory control by using the Go/No-go paradigm. The behavioral results showed that the accuracies for No-go trials were lower in the fast than in slow tempo music conditions, while the accuracies for Go trials were also lower in the fast tempo than in no music conditions. These behavioral results might indicate an impaired inhibitory control when listening to fast tempo music.

Consistent with previous studies (Nieuwenhuis et al., 2003), the present study showed larger N2 amplitudes in No-go than in Go conditions, irrespective of the type of background music. Moreover, we also observed a significant interaction effect between stimulus type and music tempo. The N2d amplitudes, obtained by subtracting the Go from the No-go conditions, were larger in the fast tempo music condition than in the three other conditions. The N2 component in the inhibitory control tasks was suggested to reflect the detection of response conflict (Nieuwenhuis et al., 2003) and also a recruitment of attentional resource for the following response inhibition (Van Veen and Carter, 2002; Yuan et al., 2012). Jodo and Kayama (1992) found that the No-go N2 amplitudes were larger under high than under low time pressure condition. The participants in the high time pressure condition were required to make Go responses within a shorter period, which thus resulted in fast responses to Go trials. Jodo and Kayama (1992) suggested that the faster responses to the Go trials could enhance the Go responses, which would be more difficult to be withheld on the appearance of the No-go trials. Thus, increased efforts were required to inhibit the Go response to No-go trials, which thus contributed to enhanced N2 amplitudes (Jodo and Kayama, 1992). In the current study, the behavioral responses to Go trials were faster in the fast tempo music condition than those in the three other conditions. This result was consistent with the previous study showing that faster responses were induced by listening to fast than to slow tempo music during a visual selective attention task (Amezcua et al., 2005). Thus, more cognitive efforts would be required to produce appropriate No-go response in a fast tempo music condition, which contributed to larger N2d amplitudes.

Moreover, consistent with previous studies (Falkenstein et al., 1999; Gajewski and Falkenstein, 2013), larger P3 amplitudes were observed for No-go trials relative to Go trials in the present study. It has been generally considered that the P3 predominantly represents motor or response inhibition (Enriquez-Geppert et al., 2010). However, we did not observe the interaction effect between stimulus type and music tempo. In other words, the P3d amplitudes were similar among the four music conditions. This finding suggested that the tempo of music did not affect the later response inhibition.

However, it should be noted that the tempo of the music is one of the potential factors for inducing emotion (Kim et al., 2018). Thus, the emotion effect induced by music tempo cannot be completely ruled out when investigating the influence of music tempo on inhibitory control and thus would form a contamination for the present study. However, the self-reported rate of music in terms of induced pleasure, arousal, and preference could rule out this possibility because there were no significant differences on arousal and preference rating among these three types of music conditions. Although a significant main effect on pleasure was observed, no significant differences were observed between medium and fast or between slow and fast tempo music conditions. Thus, the ERP effects at the N2d were more likely specific to the tempo of music rather than the induced pleasure, arousal, and preference.

Taken together, the present study, using ERPs, demonstrated an obvious effect of music tempo on inhibition control. More specifically, listening to fast music would impair an individual’s ability to monitor conflict. To our knowledge, this is the first time that the influences of music tempo on inhibitory control are directly investigated. In the future, the present findings should be replicated and verified by other experimental paradigms, especially the two-choice oddball task, which can provide the RT index of behavioral inhibitory control that the Go/No-go task does not have (Yuan et al., 2008, 2012).

## Data Availability Statement

The datasets generated for this study are available on request to the corresponding author.

## Ethics Statement

The studies involving human participants were reviewed and approved by the Ethics Committee of Hunan Normal University. The patients/participants provided their written informed consent to participate in this study.

## Author Contributions

RX and JieC designed the study. RX, JieC, CL and JiejC wrote the manuscript and carried out all data analyses. RX, CL and JiejC conducted data collection. All authors contributed to and approved the final version of the manuscript.

## Conflict of Interest

The authors declare that the research was conducted in the absence of any commercial or financial relationships that could be construed as a potential conflict of interest.
